# Rapid Point-of-Care Detection of *Dirofilaria immitis* and *Dirofilaria repens* in Canine Blood Using Two Direct Closed-Tube LAMP Assays

**DOI:** 10.3390/ani16121820

**Published:** 2026-06-12

**Authors:** Zsófia Bujtor, Tünde Földvári, Csaba Pribenszky, Ákos Jerzsele, Petra Zenke

**Affiliations:** 1 Department of Animal Breeding and Genetics, University of Veterinary Medicine Budapest, Istvan u. 2, H-1078 Budapest, Hungary; bujtor.zsofia.krisztina@student.univet.hu (Z.B.);; 2 Department of Animal Hygiene, Herd Health and Mobile Clinic, University of Veterinary Medicine Budapest, Istvan u. 2, H-1078 Budapest, Hungary; pribenszky.csaba@univet.hu; 3 Department of Pharmacology and Toxicology, University of Veterinary Medicine Budapest, Istvan u. 2, H-1078 Budapest, Hungary; jerzsele.akos@univet.hu; 4 National Laboratory of Infectious Animal Diseases, Antimicrobial Resistance, Veterinary Public Health and Food Chain Safety, University of Veterinary Medicine Budapest, H-1078 Budapest, Hungary

**Keywords:** DNA extraction, blood samples, canine dirofilariasis, loop-mediated isothermal amplification, point-of-care diagnostics, direct LAMP assays, veterinary diagnostics

## Abstract

Canine dirofilariasis, caused by *Dirofilaria immitis* and *Dirofilaria repens*, is an emerging vector-borne disease of increasing veterinary and zoonotic importance. Rapid and species-specific detection is essential for effective clinical management and epidemiological surveillance. This study aimed to develop and diagnostically evaluate two novel species-specific loop-mediated isothermal amplification (LAMP) assays for the direct detection of *D. immitis* and *D. repens* in canine whole blood, performed in parallel in separate reactions, with emphasis on simplified and potentially near-point-of-care applicability. Primers targeting mitochondrial COI and NADH gene regions were designed and validated. In silico specificity analysis against 13 filarioid species confirmed the absence of non-specific primer binding. A direct closed-tube LAMP protocol using sodium hydroxide–Chelex-100 lysis was optimized, enabling amplification without conventional DNA extraction while reducing contamination risk and processing time to under 60 min. Relative diagnostic performance was evaluated relative to quantitative real-time PCR (qPCR) results. Using purified DNA, the *D. repens* assay achieved 100% relative sensitivity and relative specificity, whereas the *D. immitis* assay showed 94.5% relative sensitivity and 100% specificity. In direct whole-blood assays, relative specificity remained 100% for both targets, while sensitivity decreased to 90.9% for *D. immitis* and 77.42% for *D. repens*, with most false-negative reactions associated with high qPCR Ct values (>30). These findings demonstrate that the proposed assays provide a rapid and practical molecular diagnostic approach with potential applicability for point-of-care veterinary testing.

## Simple Summary

Heartworm and skin worm infections in dogs, caused by *Dirofilaria* parasites, are spreading in many regions and rarely can also pose risks to humans. Early and accurate detection is important for proper treatment and for monitoring the spread of these infections. However, current laboratory methods are often time-consuming and require specialized equipment. In this study, we developed two rapid and simple diagnostic tests that can detect these parasites directly from a small amount of dog blood. The tests are based on a technique called LAMP, which allows DNA detection without complex laboratory procedures, and are performed in parallel in separate reactions to specifically identify *Dirofilaria immitis* and *Dirofilaria repens*. The method does not require traditional DNA extraction and can deliver results in less than one hour, making it potentially adaptable for use in veterinary clinics or field settings. We found that the tests were highly accurate and correctly identified most infected samples. In cases with very low levels of infection, sensitivity was slightly lower than that of standard laboratory methods. Overall, this approach provides a fast, affordable, and practical tool for detecting *Dirofilaria* infections in dogs, which may help improve animal health and support disease control efforts.

## 1. Introduction

The most common canine diseases caused by parasites of the genus *Dirofilaria* are canine heartworm disease, caused by *D. (Dirofilaria) immitis*, and canine subcutaneous dirofilariasis, caused by *D. repens*. Both filarial nematodes are transmitted by mosquitoes, acting as intermediate hosts and primarily infect dogs and other canids, while cats and humans may serve as accidental hosts. Formerly restricted to tropical regions, *D. immitis* has now established endemic foci in temperate areas worldwide [1,2,3,4,5]. In Hungary, imported cases were reported in 1982 and 2000, followed by the first autochthonous case in 2007 [6,7,8]. Its spread is associated with dog movement, climate change favoring mosquito vectors, and wildlife reservoirs such as red foxes and golden jackals [3,9,10,11].

The incidence of *D. repens* has also increased across Europe, Asia, and Africa, likely driven by globalization and climate change. In Hungary, infections were reported before 2000 [6,12]. Although typically less pathogenic in dogs than *D. immitis*, *D. repens* is of particular concern due to its higher zoonotic potential [3,4,13,14,15,16]. Clinically, *D. immitis* infection may lead to severe cardiopulmonary disease, including pulmonary hypertension and heart failure, while *D. repens* infections are usually subcutaneous but can also affect humans, occasionally involving ocular or other tissues [3,10,14,15,17,18,19,20,21].

Diagnosis relies primarily on blood-based methods. Microscopy (e.g., Knott test) is widely used but has limited sensitivity, depends on operator expertise, and cannot reliably distinguish between species or detect occult infections [10,22,23,24,25,26,27]. Antigen tests improve sensitivity for *D. immitis* but are unavailable for *D. repens* and may yield false results [25,27,28]. Imaging techniques support disease assessment, while molecular methods such as PCR enable sensitive and specific detection and species differentiation but require specialized laboratory infrastructure [23,26,27,29,30,31,32,33,34].

Loop-mediated isothermal amplification (LAMP) has emerged as a rapid and cost-effective alternative, operating at a constant temperature and producing results within one hour with minimal equipment [16,34,35,36,37,38]. Its high tolerance to inhibitors and comparable sensitivity to PCR make it suitable for field diagnostics [35,38,39,40,41,42,43]. However, conventional molecular methods require DNA extraction, which increases time and cost [43]. Direct amplification approaches, using crude samples prepared by simple lysis methods, offer faster and potentially more practical near-point-of-care testing [44,45,46,47,48].

Despite these advantages, LAMP’s high sensitivity increases the risk of contamination, which can be mitigated by single-tube closed systems [48,49]. Although LAMP has been applied to various parasitic infections, its routine use in veterinary diagnostics remains limited. Wider implementation could improve early detection and epidemiological surveillance of canine dirofilariasis [50,51,52,53,54,55].

This study aimed to develop two direct, closed-tube LAMP assays performed in parallel in separate reaction tubes for the detection of *D. immitis* and *D. repens* in canine blood without DNA extraction. The assays were evaluated against quantitative real-time PCR (qPCR) results obtained from purified DNA, used as a molecular comparator.

## 2. Materials and Methods

### 2.1. LAMP Primer Design

Sequences of the COI (cytochrome c oxidase) and NADH (nicotinamide adenine dinucleotide dehydrogenase, also referred to as ND) genes of the two target *Dirofilaria* species were retrieved from the National Center for Biotechnology Information (NCBI) GenBank database (https://www.ncbi.nlm.nih.gov/genbank/, accessed on 5 August 2025) (Tables S1 and S2). The selected sequences were aligned by ClustalW, implemented in MEGA 6.0 (Molecular Evolutionary Genetics Analysis) software [56] to identify conserved and species-specific regions, as well as single-nucleotide differences between *D. immitis* and *D. repens*. For each gene, seven primer sets were designed using the PrimerExplorerV5 online tool (https://primerexplorer.eiken.co.jp/lampv5e/, accessed on 28 February 2026). Some primer sets included automatically generated loop primers in addition to the four essential LAMP primers (Tables S1 and S2). Additionally, for each gene, one previously published primer set was included in the study for comparative purposes [16,34].

### 2.2. Preliminary Tests of the Designed Primer Sets

An in silico specificity analysis was performed first to assess the species specificity of the 16 designed primer sets for *D. immitis* and *D. repens* (Tables S1 and S2).

Subsequently, in vitro analyses were conducted using 40 DNA isolates obtained from canine (*Canis lupus familiaris*) blood samples. These samples were collected from dogs suspected of infection with one or both *Dirofilaria* species (*D. immitis* and *D. repens*) at veterinary clinics across Hungary between 2023 and 2024. Samples represented a convenience cohort submitted for routine veterinary diagnostic testing. Although most dogs tested positive using on-site diagnostic methods (ELISA and/or the Knott test), a smaller number of negative samples were also included; however, these preliminary results were excluded from the study due to inconsistencies in reporting and methodology among the participating clinics. Blood samples were collected into EDTA-anticoagulated tubes and stored at −20 °C until further laboratory analyses. Genomic DNA was extracted using the FavorPrep™ Tissue Genomic DNA Extraction Mini Kit (Favorgen Biotech, Ping-Tung, Taiwan) according to the manufacturer’s instructions. DNA concentration was measured using a Qubit 2.0 Fluorometer (Life Technologies Corporation, Carlsbad, CA, USA), and DNA quality was assessed by electrophoresis on a 1% agarose gel stained with GelGreen™ Nucleic Acid Gel Stain (Biotium, Fremont, CA, USA).

All designed and previously published primer sets (16 in total) were tested in duplicate, with each reaction performed in parallel, resulting in a total of 2 × 640 LAMP reactions. Duplicate reactions were evaluated independently. In cases of discordant duplicate results, the corresponding samples were retested, and the final false-positive and false-negative counts reported in Section 3.1 represent concordant outcomes obtained after repeated testing. LAMP reactions were prepared separately for each primer set in a final volume of 5 μL, containing 3 μL of 2× Isothermal Mastermix (ISO-004, OptiGene, Horsham, West Sussex, UK), 0.5 μL of primer mix (FIP/BIP primers, 40 μM stock; F3/B3 primers, 5 μM stock; LF/LB primers, 20 μM stock, included only in four *D. immitis* and three *D. repens* primer sets), 0.5 μL of Betaine (5 M stock; ThermoFisher Scientific, Waltham, MA, USA), and 1 μL of DNA template. Reactions were incubated in PCR-tubes in a pre-heated thermocycler set to 65 °C for 45 min. Non-template controls (NTCs) were included in each run.

Amplification was assessed qualitatively using two end-point methods: (a) 1 μL of GelRed™ Nucleic Acid Gel Stain (10,000× stock; Biotium, Fremont, CA, USA), diluted 1:10 (*v*/*v*) in nuclease-free water, was added directly to each reaction tube and visualized under blue-light using a Glite 900 BW Gel Scanner (Pacific Image Electronics Co., Ltd., New Taipei City, Taiwan); and (b) amplified products were electrophoresed on a 2% agarose gel stained with the same GelRed™ Nucleic Acid Gel Stain for 20 min and visualized under blue-light using the same scanner.

Subsequently, to confirm the presence or absence of *D. immitis* and *D. repens*, blood-derived DNA isolates were additionally screened by quantitative real-time PCR, as previously described [11,30]. Based on qPCR comparison, iCOI-44 (*D. immitis*) and rND5-1 (*D. repens*) were selected from several candidate primer sets for direct LAMP testing. Primer selection was based on preliminary in vitro performance, including concordance with qPCR results and the occurrence of false-positive and false-negative reactions. Amplification kinetics, fluorescence intensity, and inter-run reproducibility were not quantitatively evaluated during primer screening.

### 2.3. Direct LAMP Assays Using EDTA Whole Blood

**Figure 1:**
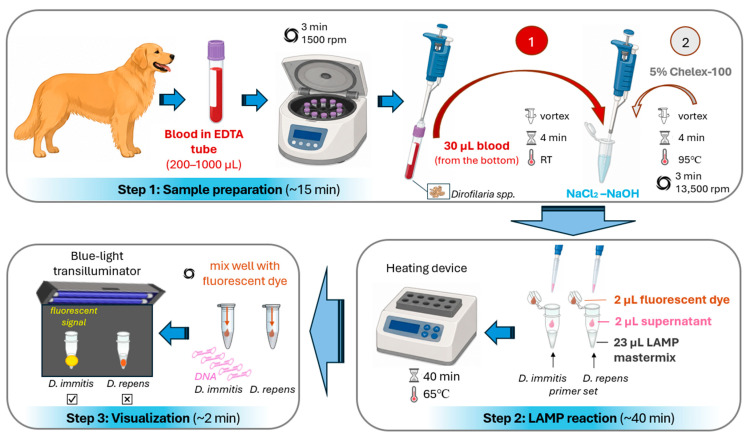
Schematic representation of the workflow of LAMP assays for the detection of *Dirofilaria* spp. in blood samples. Sample preparation is performed using EDTA blood collected from dogs suspected of having dirofilariasis. Crude DNA samples are subjected to LAMP for 40 min, followed by post-amplification fluorescence detection. The total turnaround time from whole blood sample to visual result was approximately 55–60 min, including ~15 min hands-on processing time and 40 min amplification time.

Following a rapid 10-min chemical pretreatment, direct LAMP assays were developed from whole-blood samples as described below and in Figure 1. Whole blood was centrifuged at 1500 rpm for 3 min, after which 30 µL of blood was collected from the bottom of the tube and mixed with 30 µL of 0.3 M NaCl and 150 µL of 25 mM NaOH. Samples were incubated at room temperature for 4 min, followed by the addition of 210 µL of 5% Chelex® 100 (Bio-Rad Laboratories, Hercules, CA, USA) suspension and thorough vortexing. After incubation at 95 °C for 4 min, samples were centrifuged at 13,000× *g* for 3 min, and 2 µL of the supernatant was used as template for the LAMP reaction.

Reactions were performed in a final volume of 25 µL, containing 15 µL of 2× Isothermal Mastermix (ISO-004, OptiGene, Horsham, West Sussex, UK), 2.5 µL of primer mix (FIP/BIP primers, 40 µM stock; F3/B3 primers, 5 µM stock; LF/LB primers, 20 µM stock) (Table 1), 5.5 µL of nuclease-free water, and 2 µL of DNA template. The selected primer sets for *D. immitis* and *D. repens* were applied in separate, parallel reactions for each target species. Negative blood controls and non-template controls were included in each run.

For closed-tube detection, 2 µL of GelGreen™ Nucleic Acid Gel Stain (10,000× stock; Biotium, Fremont, CA, USA), diluted 1:10 (*v*/*v*) in nuclease-free water, was placed into the cap of each reaction tube during setup, and the tubes were carefully sealed. Amplification was carried out in a preheated thermocycler at 65 °C for 40 min, followed by incubation at 80 °C for 2 min to terminate the reaction. After amplification, a brief centrifugation step was applied to mix the dye with the reaction product, and fluorescence was immediately visualized under blue-light illumination using a Glite 900 BW Gel Scanner. Visual interpretation of fluorescence results was performed independently by two operators blinded to the qPCR results.

### 2.4. Validation Study

An in silico specificity analysis was performed by comparing the primer sequences used for direct amplification of *D. immitis* (iCOI-44) and *D. repens* (rND5-1) with the full-length or partial COI and ND5 sequences from other parasite worm species typical in dogs. Sequence comparisons were conducted using the online BLAST tool provided by the National Center for Biotechnology Information (NCBI) [57]. The nucleotide sequences corresponding to F1c and F2 (comprising the FIP primer), as well as B1c and B2 (comprising the BIP primer), were separated and evaluated individually. Matches were considered significant when at least 95% of the query oligonucleotide length showed ≥90% sequence identity to the target sequence, with no mismatches present within the last three nucleotides at the 3′ terminus.

In vitro specificity and sensitivity analyses of the direct amplification method were conducted using a total of 90 canine blood samples, comprising the previously described samples and an additional 50 samples collected during the same period, at similar locations, and using identical sampling procedures. The initial 40-sample cohort was used for preliminary in vitro primer screening and selection of the most suitable assays, after which the selected *D. immitis* and *D. repens* primer sets were further evaluated on the complete 90-sample validation cohort using both purified DNA and direct amplification from EDTA whole blood. Qualitative results were evaluated according to the direct LAMP protocol.

To confirm the presence or absence of *D. immitis* and *D. repens*, DNA was isolated from the additional 50 samples and subsequently screened by quantitative real-time PCR, as described in Section 2.2. The relative diagnostic performance of the developed direct LAMP assays was evaluated against quantitative real-time PCR (qPCR) results obtained from the corresponding blood samples (*n* = 90).

The complete 90-sample dataset was used to estimate overall assay performance. Because the initial 40-sample cohort contributed to primer selection, diagnostic performance was additionally evaluated using the independent set of 50 subsequently collected samples. Relative diagnostic performance was evaluated using standard definitions of sensitivity, specificity, and accuracy based on true positive (TP), true negative (TN), false positive (FP), and false negative (FN) results. Relative sensitivity was defined as TP/(TP + FN), representing the proportion of infected samples correctly identified by the assay. Accordingly, it reflects the probability that *D. immitis*-positive samples yielded positive results in the corresponding LAMP reactions, and similarly, that samples infected with *D. repens* tested positive in the respective assays. Relative specificity was defined as TN/(TN + FP), representing the proportion of target-negative samples correctly identified as negative by the corresponding assay. Accordingly, specificity calculations included all samples negative for the target species, including samples positive for the alternate *Dirofilaria* species as well as uninfected control samples. Accuracy was defined as (TP + TN)/(TP + TN + FP + FN). Ninety-five percent confidence intervals (95% CI) for relative sensitivity, specificity, and accuracy were calculated using the Clopper–Pearson exact binomial method [58].

Sanger sequencing was applied to representative PCR products from two *D. immitis*–positive and two *D. repens*–positive samples to definitively confirm whether the targeted COI and ND5 regions were amplified from the dog blood DNA isolates. Both markers were amplified with locus-specific F3/B3 primers in singleplex PCR reactions (Table 1). The PCR reactions (25 μL in volume) consisted of 5 μL of DreamTaq™ Green PCR Master Mix (ThermoFisher Scientific, Waltham, MA, USA), 0.5 μM of F3, and 0.5 μM of B3 primers, 1 ng DNA template, and PCR-grade H_2_O to volume. PCR was carried out in an Applied Biosystems 2720 Thermal Cycler (Applied Biosystems, Foster City, CA, USA) with the following conditions: an initial 95 °C for 10 s. followed by 36 cycles of 20 s at 94 °C, annealing of 20 s at 54 °C, and 20 s at 72 °C. Qualitative assessments of singleplex amplifications were conducted using 2% agarose gel stained with GelRed^TM^ Nucleic Acid Gel Stain (Biotium, Fremont, CA, USA).

Amplification products were purified using GenEluteTM PCR Clean-Up Kit (Sigma–Aldrich, St. Louis, MO, USA). Both DNA strands were sequenced using the BigDye^®^ Terminator v.1.1 Cycle Sequencing Kit (Thermo Fisher Scientific, Waltham, MA, USA) following the manufacturer’s recommendations. PCR amplicons were sequenced by an ABI Prism 3130XL Genetic Analyzer (Applied Biosystems, Waltham, MA, USA), according to the manufacturer’s guidelines. Sequence analyses were performed using Sequencing Analysis Software 5.1 (Applied Biosystems, Waltham, MA, USA). Sequence alignment was conducted with Sequencher™ 5.4.6 software (Gene Codes Corp., Ann Arbor, MI, USA), and nucleotide sequences were compared with reference sequences available in GenBank using BLAST.

## 3. Results

### 3.1. Selection of Primer Sets

The primer sets were first analyzed in silico by comparing nucleotide differences between the DNA sequences of the two *Dirofilaria* species to minimize false-positive results and cross-reactivity (Tables S1 and S2).

The in vitro performance of the 16 primer sets using purified DNA varied considerably, as summarized in Table 2. The performance of the LAMP primer sets was evaluated against quantitative real-time PCR (qPCR) results obtained during parallel testing (Table S3). Primer sets were tested both with and without loop primers, and the inclusion of loop primers consistently improved amplification efficiency. For *D. immitis*, primer sets iCOI-1, iCOI-44, iND1-1, and iND1-22 showed the lowest numbers of false-positive and false-negative reactions during preliminary in vitro testing, while for *D. repens*, sets rCOI-2, rCOI-4, and rND5-1 demonstrated the highest diagnostic concordance with qPCR results and reproducibility during repeated testing. False-positive and false-negative counts presented in Table 2 represent final concordant sample-level outcomes obtained after duplicate testing and repeat analysis of discordant reactions. Based primarily on diagnostic concordance with qPCR results and the absence of false-positive reactions during preliminary testing, primer sets iCOI-44 (for *D. immitis*) and rND5-1 (for *D. repens*) were selected for the subsequent direct amplification experiments (Table 1).

When DNA sequences from non-*Dirofilaria* parasite species were analyzed in silico, neither the COI nor the ND5 gene regions contained potential annealing sites for more than three of the six oligonucleotides comprising the LAMP primer set. Furthermore, no simultaneous matches were observed for the F2 and B2 primers, which are responsible for initiating the LAMP process, within the COI or ND5 genes of any non-target species analyzed (Table 3). These findings support the high predicted species specificity of the designed primer sets.

As part of primer testing, traditional monoplex PCR amplification of both markers in the two examined worm species produced gene segments of the expected lengths. The PCR products were confirmed by sequencing, and the resulting nucleotide sequences were compared with entries in GenBank. Complete (100%) homology was observed with sequences corresponding to the respective species and gene segments. The sequences have been deposited in NCBI GenBank under accession numbers PZ229228 (COI gene segment of *D. immitis*) and PZ231385 (ND5 gene segment of *D. repens*).

### 3.2. Direct Amplification from EDTA Whole Blood

Using the NaOH–Chelex-100 lysis method and the selected primer sets, the direct LAMP assay correctly identified 79 of 90 blood samples at the sample level and 168 of 180 reactions at the reaction level when compared with previously obtained qPCR results from purified DNA (Table 4). At the target-species level, no false-positive reactions were observed for either assay; however, twelve false-negative reactions occurred, predominantly affecting *D. repens*. Most false-negative samples had qPCR cycle threshold (Ct) values above 30, indicating low parasite concentrations. In co-infected samples, classification was considered incomplete when one target species was successfully detected while the second target was missed. In three co-infected samples (*D. immitis* and *D. repens* positive), the direct LAMP assay successfully detected *D. immitis* but failed to amplify *D. repens*.

### 3.3. Visual Detection and Relative Diagnostic Performance

**Figure 2:**
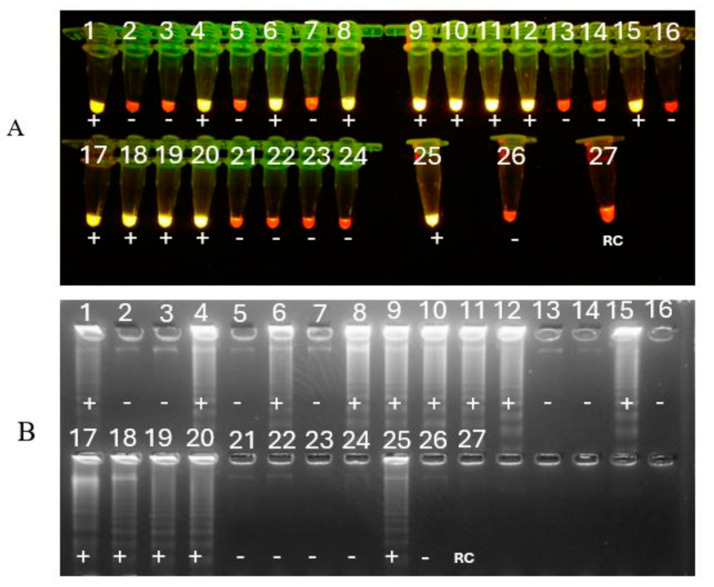
Detection of *Dirofilaria immitis* in 27 samples using the iCOI-44 primer set. +: true positive; −: true negative; RC: reagent control. (**A**) Direct visualization after mixing with intercalating dye under blue-light illumination. (**B**) Confirmation of amplification results by agarose gel electrophoresis.

Using GelGreen™ fluorescent dye under blue-light illumination, positive samples exhibited green fluorescence, whereas negative controls and false-negative samples retained the original orange coloration, allowing clear visual discrimination in both detection approaches (Figure 2). No discrepancies between the two independent readers were observed during visual interpretation of fluorescence results.

Relative diagnostic performance was evaluated using standard definitions of sensitivity, specificity, and accuracy based on true positive (TP), true negative (TN), false positive (FP), and false negative (FN) results. Performance values were calculated separately for each target species and evaluated relative to qPCR results obtained from purified DNA. When using purified DNA, the selected primer sets demonstrated high relative diagnostic performance across 90 samples. To assess performance independently of the primer-selection cohort, diagnostic performance estimates for the additional 50-sample validation subset were also calculated and are presented separately in Supplementary Table S5. The results showed no substantial differences compared with those obtained for the complete 90-sample dataset. The *D. repens* assay achieved a relative sensitivity of 100% (31/31; 95% CI: 88.8–100%), and a relative specificity of 100% (59/59; 95% CI: 93.9–100%) within the tested sample set, and an overall accuracy of 100% (90/90; 95% CI: 96.0–100%). The *D. immitis* assay yielded false-negative results in 3.33% of samples, corresponding to a relative sensitivity of 94.5% (35/37; 95% CI: 81.9–99.3%), a relative specificity of 100% (53/53; 95% CI: 93.3–100%), and an overall relative accuracy of 96.67% (87/90; 95% CI: 90.6–99.3%).

In contrast, when applying the direct LAMP assays, a slight reduction in sensitivity was observed, while specificity remained unchanged. The direct *D. immitis* assay produced false-negative results in 5.55% of samples, corresponding to a relative sensitivity of 90.9% (30/33; 95% CI: 75.7–98.1%), a relative specificity of 100% (57/57; 95% CI: 93.7–100%), and an overall accuracy of 94.44% (85/90; 95% CI: 87.5–98.2%). The direct *D. repens* assay showed a false-negative rate of 7.77%, corresponding to a relative sensitivity of 77.42% (24/31; 95% CI: 58.9–90.4%), a relative specificity of 100% (59/59; 95% CI: 93.9–100%), and an overall accuracy of 92.22% (83/90; 95% CI: 84.6–96.8%). At the sample level, co-infected samples in which only one target species was detected were classified as partially discordant relative to qPCR results (Table S4).

## 4. Discussion

Primer design is a key determinant of LAMP assay performance, as the method relies on the coordinated binding of multiple primers targeting distinct regions of the template. Even minor mismatches can affect amplification efficiency and specificity; therefore, in silico evaluation is essential, but must be complemented by in vitro validation to confirm assay performance. In agreement with this, our results revealed substantial variability among the designed primer sets despite similar in silico predictions, with only a subset demonstrating optimal relative diagnostic performance. However, primer selection was primarily based on concordance with qPCR results and false reaction frequencies, while amplification kinetics, fluorescence intensity, and inter-assay reproducibility were not systematically quantified. These findings highlight the necessity of combined in silico and in vitro evaluation for the development of reliable LAMP-based diagnostic assays [59,60,61,62,63,64].

Minimizing carryover contamination remains a critical challenge in highly sensitive nucleic acid amplification methods. Single closed-tube LAMP systems address this limitation by enabling amplification and detection within a sealed reaction tube, reducing the risk of amplicon aerosolization and false-positive results. Such systems are well suited for species-specific detection of *D. immitis* and *D. repens*, and in this study were successfully combined with direct LAMP assays from whole blood to enable rapid, potentially field-adaptable diagnostics.

Several detection strategies have been integrated into closed-tube LAMP assays, including intercalating fluorescent dyes, metal ion indicators, pH-sensitive colorimetric reagents, and turbidity measurement based on magnesium pyrophosphate precipitation, allowing real-time or endpoint detection without opening the reaction tube [60,64,65,66]. In the present study, fluorescence-based detection further supported assay robustness, as no cross-contamination or false-positive results attributable to amplicon carryover were observed.

Direct LAMP from unprocessed clinical samples, including whole blood, has been widely demonstrated, improving applicability in point-of-care settings [46,48,49,50,51]. Simplified preparation methods, such as boiling, detergent-based lysis (e.g., Triton X-100), or filter paper-based approaches, enable rapid DNA release while reducing processing time, equipment requirements, and costs [67,68,69,70,71,72]. In addition, most LAMP detection formats—including fluorescence, pH-based colorimetry, and turbidity measurement—can be performed directly in a closed tube without additional steps [48,49,63,64,65]. These features collectively enhance the practicality of LAMP for simplified or near-point-of-care applications [44,46,72,73].

Both assays developed in this study provide a rapid and simple alternative to conventional molecular diagnostics for detecting *Dirofilaria* species in canine blood. When using purified DNA, the selected primer sets demonstrated high relative diagnostic performance, with complete relative sensitivity achieved for the *D. repens* assay and 94.5% relative sensitivity for the *D. immitis* assay. Considerable variability among the initially designed primer sets further underscored the importance of rigorous primer validation. The NaOH–Chelex-100-based direct LAMP assay enabled DNA release without conventional extraction, significantly reducing processing time, cost, and equipment requirements, thereby improving applicability in simplified laboratory or near-point-of-care settings. In veterinary practice, the proposed direct closed-tube LAMP workflow may serve as a rapid complementary molecular diagnostic tool alongside routinely applied antigen testing and the Knott test, particularly when rapid species-specific confirmation is required.

However, when applied directly to whole blood, a moderate reduction in relative diagnostic sensitivity was observed compared with quantitative real-time PCR. This decrease is likely attributable to residual inhibitory substances, incomplete cell lysis, unequal DNA release, or differences in primer-template interaction efficiency under direct amplification conditions. Although many false-negative reactions were associated with high qPCR Ct values indicating low parasite DNA concentrations, several missed *D. repens* detections also occurred at moderate Ct values, suggesting that factors other than low target abundance may have contributed to reduced amplification efficiency. These findings are consistent with previous studies demonstrating that, although LAMP assays are rapid, robust, and more tolerant to inhibitors, their analytical sensitivity may be lower than that of quantitative real-time PCR or nested PCR, particularly in samples with low target DNA concentrations or when direct amplification is applied [74,75,76]. In addition, reduced detection of *D. repens* in co-infected (Dir) samples suggests potential differences in amplification efficiency or template availability under direct conditions. In several co-infected samples, relatively high qPCR Ct values for *D. repens* combined with substantially lower Ct values for *D. immitis* indicated marked differences in target DNA abundance, which may have reduced *D. repens* detection efficiency due to incomplete lysis, unequal DNA release, or preferential primer binding to the more abundant *D. immitis* DNA under direct amplification conditions. At the sample level, several co-infected samples showed only partial concordance with qPCR results because one target species was successfully detected while the second target remained undetected. Therefore, although the developed direct closed-tube LAMP assays are suitable for rapid point-of-care diagnostics, samples with very low parasite DNA concentrations may still require confirmation by more sensitive laboratory-based molecular methods. This limitation should be considered particularly during routine clinical screening or in suspected occult infections with low circulating microfilarial burden. LAMP-based detection may be less sensitive in cases of occult dirofilariasis, in which adult worms are present but microfilariae are absent from the bloodstream. This condition may occur in infections involving only immature worms or single-sex populations, preventing microfilariae production. In addition, prophylactic treatments and host immune responses can eliminate circulating microfilariae, resulting in occult infection [10,24,25,26]. Another limitation of the current direct-LAMP workflow is the absence of an internal amplification control. Consequently, amplification failure caused by inhibitory substances or other reaction-associated factors in individual samples cannot be readily distinguished from true negative results or very low target DNA concentrations in individual samples.

Despite these limitations, both assays maintained high relative diagnostic specificity within the tested sample set, with no false-positive results observed relative to qPCR. However, since no in vitro cross-reactivity testing was performed against closely related non-target filarioid species or other clinically relevant bloodborne parasites, additional specificity studies would be valuable to further confirm assay performance under broader diagnostic conditions. Nevertheless, the rapid turnaround time and operational simplicity support their potential use in simplified laboratory or near-point-of-care settings following further validation. Further optimization of the lysis protocol and reaction conditions—such as improving inhibitor tolerance or enhancing DNA release efficiency—may improve diagnostic sensitivity and ensure more reliable detection in low-parasite-load samples. Future multicenter studies and inter-operator reproducibility assessments will also be important to further validate the robustness and field applicability of the proposed assays under routine veterinary practice conditions. In addition, multiplex detection within a single reaction may be achievable by applying differently labeled fluorescent probes or primers, allowing simultaneous discrimination of *D. immitis* and *D. repens* in a closed-tube format, as demonstrated in previous LAMP-based multiplex approaches [76,77,78]. Overall, the method represents a promising approach for rapid and cost-effective detection of canine dirofilariasis in simplified laboratory or near-point-of-care settings.

## 5. Conclusions

The developed LAMP assays represent a rapid, simple, and reliable approach for the detection of *Dirofilaria immitis* and *D. repens* in canine blood. The integration of a single closed-tube system with direct LAMP reaction significantly enhances its potential applicability in simplified laboratory and near-point-of-care settings by minimizing contamination risk and eliminating the need for DNA extraction. Consequently, such systems represent a key advancement toward safe, reliable, and potentially field-adaptable molecular diagnostic platforms. While a slight reduction in sensitivity was observed compared with qPCR, the method demonstrated high specificity and overall accuracy. With further optimization, this approach has strong potential as a practical and cost-effective diagnostic tool for veterinary and epidemiological use, effectively bridging the gap between laboratory-based diagnostics and field applicability.

## Figures and Tables

**Table 1:** Primer sequences and concentrations of LAMP primers used for direct amplification of *Dirofilaria immitis* (iCOI-44) and *D. repens* (rND5-1).

Primer Name	Type	Sequence (5′-3′)	Primer Stock Concentration (μM)
iCOI-44 F3	Forward outer	TTTTGGACATCCTGAGGTT	5.0
iCOI-44 B3	Backward outer	GCAGCACTAAAATAAGTACGA	5.0
iCOI-44 FIP	Forward inner (F1c + F2)	GGCCAAACAAACGATCCTTATCAG-TATTTTACCGGTGTTTGGGAT	40.0
iCOI-44 BIP	Backward inner (B1c + B2)	TGACTTTTGCTTCTATTTGGATTGC-GTATCAATATCCAAACCAGCT	40.0
iCOI-44 LB	Loop backward	TATTGGGGACTTCTGTTTGGGGT	10.0
rND5-1 F3	Forward outer	CTTTTGTTAAGGGTGGTCAG	5.0
rND5-1 B3	Backward outer	CAAAAACCAGAAAAAACCAAAGT	5.0
rND5-1 FIP	Forward inner (F1c + F2)	ACTATGAACCAAACAACTAACAGGA-TATCCTTTTGGTAGTTGGCTT	40.0
rND5-1 BIP	Backward inner (B1c + B2)	GTTACTGCTGGTGTTATGTTAATGG-ACAAAAGACAACACATCAGAA	40.0
rND5-1 LF	Loop forward	GGGGCAGCCATAGCTTTAGG	10.0

**Table 2:** Preliminary in vitro performance of the 16 LAMP primer sets on DNA isolates obtained from canine blood samples (*n* = 40). Primer sets were tested in duplicate, and false-positive/false-negative counts represent final concordant sample-level outcomes after repeat testing of discordant duplicate reactions.

*Dirofilaria immitis*			*Dirofilaria repens*		
Primer Sets	False Negative	False Positive	Primer Sets	False Negative	False Positive
iCOI-1	0	0	rCOI-2	0	0
iCOI-2	0	4	rCOI-4	0	0
iCOI-15	16	0	rCOI-14	0	6
iCOI-32	1	0	rCOI-18	8	2
iCOI-44	0	0	rCOI-27	19	0
iND1-1	0	0	rCOI-36	14	5
iND1-11	0	0	rCOI-44	19	1
iND1-22	0	0	rND5-1	0	0

**Table 3:** Parasite species included in the in silico specificity analysis using the primer sequences of the two LAMP primer sets, iCOI-44 for *Dirofilaria immitis* and rND5-1 for *D. repens*, developed for the direct amplification assay. +: oligonucleotide showing partial sequence homology with the target gene region; −: no sequence homology detected with the target gene region.

Parasite	GenBank Accession Number	In Silico Primer Test													
		iCOI-44							rND5-1						
		F3	B3	F1	F2	B1	B2	LB	F3	B3	F1	F2	B1	B2	LF
*Dirofilaria immitis*	NC_005305	+	+	+	+	+	+	+	+	−	+	−	+	−	−
*Dirofilaria repens*	NC_029975	+	−	−	−	−	+	−	+	+	+	+	+	+	+
*Acanthocheilonema reconditum*	AJ544876	+	−	−	−	+	−	−	−	−	−	−	−	−	−
*Acanthocheilonema dracunculoides*	MG797680	−	−	−	−	−	−	−	−	−	−	−	−	−	−
*Brugia malayi*	MT149211	−	+	−	−	+	+	−	−	−	+	−	−	+	−
*Brugia timori*	AP017686	−	+	−	−	−	+	−	−	−	+	−	+	+	−
*Angiostrongylus vasorum*	NC_018602	−	−	−	−	−	−	−	−	−	−	−	−	−	−
*Anaplasma phagocytophilum*	NZ_CP166491	−	−	−	−	−	−	−	−	−	−	−	−	−	−
*Anaplasma platys*	CP046391.1	−	−	−	−	−	−	−	−	−	−	−	−	−	−
*Ehrlichia canis*	NZ_CP085277	−	−	−	−	−	−	−	−	−	−	−	−	−	−
*Ehrlichia ewingii*	OP067658.1	−	−	−	−	−	−	−	−	−	−	−	−	−	−
*Babesia canis*	PV059163.1	−	−	−	−	−	−	−	−	−	−	−	−	−	−
*Wolbachia pipientis*	NZ_AP038754.1	−	−	−	−	−	−	−	−	−	−	−	−	−	−

**Table 4:** Test results on *Dirofilaria immitis* (Di) and *Dirofilaria repens* (Dr) obtained by real-time PCR (qPCR) and LAMP methods, where qPCR was performed on purified DNA, while LAMP assays were conducted both on purified DNA and by a direct amplification approach from canine whole blood without DNA extraction. (Ct: cycle threshold, +: true positive, −: true negative, background shading: false result).

Sample ID	qPCR Ct		LAMP on DNA		Direct LAMP on Blood		Sample ID	qPCR CT		LAMP on DNA		Direct LAMP on Blood	
	*Di*	*Dr*	*Di*	*Dr*	*Di*	*Dr*		*Di*	*Dr*	*Di*	*Dr*	*Di*	*Dr*
**Di1**	28	−	+	−	+	−	**Dr7**	−	25	−	+	−	+
**Di2**	33	−	+	−	+	−	**Dr8**	−	23	−	+	−	+
**Di3**	33	−	+	−	+	−	**Dr9**	−	21	−	+	−	+
**Di4**	26	−	+	−	+	−	**Dr10**	−	21	−	+	−	+
**Di5**	28	−	+	−	+	−	**Dr11**	−	27	−	+	−	−
**Di6**	26	−	+	−	+	−	**Dr12**	−	28	−	+	−	−
**Di7**	33	−	+	−	+	−	**Dr13**	−	24	−	+	−	+
**Di8**	41	−	−	−	−	−	**Dr14**	−	20	−	+	−	+
**Di9**	24	−	+	−	+	−	**Dr15**	−	23	−	+	−	+
**Di10**	28	−	+	−	+	−	**Dir1**	24	35	+	+	+	+
**Di11**	20	−	+	−	+	−	**Dir2**	30	35	+	+	+	+
**Di12**	29	−	+	−	+	−	**Dir3**	27	40	+	+	+	−
**Di13**	30	−	+	−	+	−	**Dir4**	27	29	+	+	+	+
**Di14**	30	−	+	−	+	−	**Dir5**	26	35	+	+	+	−
**Di15**	26	−	+	−	+	−	**Dir6**	36	30	+	+	+	+
**Di16**	34	−	-	−	−	−	**Dir7**	34	26	−	+	−	−
**Di17**	31	−	+	−	+	−	**Dir8**	20	33	+	+	+	+
**Di18**	26	−	+	−	+	−	**Dir9**	23	22	+	+	+	+
**Di19**	28	−	+	−	+	−	**Dir10**	24	32	+	+	+	+
**Di20**	29	−	+	−	+	−	**Dir11**	25	28	+	+	+	+
**Di21**	30	−	+	−	+	−	**Dir12**	21	37	+	+	+	−
**Di22**	23	−	+	−	+	−	**Dir13**	29	26	+	+	+	+
**Di23**	22	−	+	−	+	−	**Dir14**	24	24	+	+	+	+
**Di24**	26	−	+	−	+	−	**Dir15**	24	24	+	+	+	+
**Di25**	24	−	+	−	+	−	**Dir16**	24	27	+	+	+	+
**Di26**	24	−	+	−	+	−	**D1**	−	−	−	−	−	−
**Di27**	24	−	+	−	+	−	**D2**	−	−	−	−	−	−
**Di28**	33	−	+	−	−	−	**D3**	−	−	−	−	−	−
**Di29**	27	−	+	−	−	−	**D4**	−	−	−	−	−	−
**Di30**	24	−	+	−	+	−	**D5**	−	−	−	−	−	−
**Di31**	26	−	+	−	+	−	**D6**	−	−	−	−	−	−
**Di32**	27	−	+	−	+	−	**D7**	−	−	−	−	−	−
**Di33**	24	−	+	−	+	−	**D8**	−	−	−	−	−	−
**Di34**	24	−	+	−	+	−	**D9**	−	−	−	−	−	−
**Di35**	26	−	+	−	+	−	**D10**	−	−	−	−	−	−
**Di36**	25	−	+	−	+	−	**D11**	−	−	−	−	−	−
**Di37**	22	−	+	−	+	−	**D12**	−	−	−	−	−	−
**Di38**	31	−	+	−	+	−	**D13**	−	−	−	−	−	−
**Di39**	26	−	+	−	+	-	**D14**	−	−	−	−	−	−
**Dr1**	−	26	−	+	−	−	**D15**	−	−	−	−	−	−
**Dr2**	−	27	−	+	−	+	**D16**	−	−	−	−	−	−
**Dr3**	−	22	−	+	−	+	**D17**	−	−	−	−	−	−
**Dr4**	−	23	−	+	−	+	**D18**	−	−	−	−	−	−
**Dr5**	−	25	-	+	−	+	**D19**	−	−	−	−	−	−
**Dr6**	−	26	−	+	−	+	**D20**	−	−	−	−	−	−

## Data Availability

The data will be available from the corresponding author upon request.

## Supplementary Materials

The following supporting information can be downloaded at: https://www.mdpi.com/article/10.3390/ani16121820/s1, Figure S1: Schematic representation of the loop-mediated isothermal amplification (LAMP) mechanism and primer binding sites on the target DNA sequence. Table S1: LAMP primer sets designed and analyzed in silico for the detection of *Dirofilaria* (*D.*) *immitis*. Table S2: LAMP primer sets designed and analyzed in silico for the detection of *Dirofilaria* (*D.*) *repens*. Table S3: Results of in vitro testing of fourteen newly designed and two previously published (iCOI-2 and rCOI-2) LAMP primer sets for the detection of *Dirofilaria immitis* and *D. repens* using DNA isolates (*n* = 40) from canine blood samples. Table S4. Sample-level and reaction-level concordance of purified DNA and direct blood LAMP assays relative to qPCR results. Table S5. Diagnostic performance of the selected LAMP assays (iCOI-44 for *Dirofilaria immitis* and rND5-1 for *Dirofilaria repens*) in the independent 50-DNA sample validation cohort.

## Abbreviations

The following abbreviations are used in this manuscript:

BIP	Backward inner primer
BLAST	Basic Local Alignment Search Tool
COI	Cytochrome c oxidase subunit I
Ct	Cycle threshold
D.	*Dirofilaria*
DNA	Deoxyribonucleic acid
EDTA	Ethylenediaminetetraacetic acid
FIP	Forward inner primer
FN	False negative
FP	False positive
F3	Forward outer primer
B3	Backward outer primer
LAMP	Loop-mediated isothermal amplification
LF	Loop forward primer
LB	Loop backward primer
MEGA	Molecular Evolutionary Genetics Analysis
NaCl	Sodium chloride
NaOH	Sodium hydroxide
NCBI	National Center for Biotechnology Information
ND/ND5	NADH dehydrogenase (subunit 5)
NTC	Non-template control
PCR	Polymerase chain reaction
POC	Point-of-care
qPCR	Quantitative real-time polymerase chain reaction
TN	True negative
TP	True positive

## References

[1] Genchi C., Kramer L.H., Rivasi F. (2011). Dirofilarial Infections in Europe. Vector Borne Zoonotic Dis..

[2] Morchón R., Carretón E., González-Miguel J., Mellado-Hernández I. (2012). Heartworm Disease (*Dirofilaria immitis*) and Their Vectors in Europe—New Distribution Trends. Front. Physiol..

[3] Farkas R., Mag V., Gyurkovszky M., Takács N., Vörös K., Solymosi N. (2020). The Current Situation of Canine Dirofilariosis in Hungary. Parasitol. Res..

[4] Fuehrer H.P., Morelli S., Unterköfler M.S., Bajer A., Bakran-Lebl K., Dwużnik-Szarek D., Farkas R., Grandi G., Heddergott M., Jokelainen P. (2021). *Dirofilaria* spp. and *Angiostrongylus vasorum*: Current Risk of Spreading in Central and Northern Europe. Pathogens.

[5] Karancsi Z., Mazzag B., Bársony G., Szántó M., Jerzsele A. (2024). A szívférgesség megelőzésének és kezelésének helyzete a magyarországi állatorvosi praxisokban. Magy. Allatorvosok Lapja.

[6] Boros G., Janisch M., Sebestyén G. (1982). *Dirofilaria immitis* kutyában. Magy. Allatorvosok Lapja.

[7] Jacsó O., Mándoki M., Majoros G., Pétsch M., Mortarino M., Genchi C., Fok É. (2009). First Autochthonous *Dirofilaria immitis* (Leidy, 1856) Infection in a Dog in Hungary. Helminthologia.

[8] Vörös K., Kiss G., Baska F., Bagdi N., Széll Z. (2000). Szívférgesség kutyában. Magy. Allatorvosok Lapja.

[9] Farkas R., Gyurkovszky M., Lukács Z., Aladics B., Solymosi N. (2014). Seroprevalence of Some Vector-Borne Infections of Dogs in Hungary. Vector Borne Zoonotic Dis..

[10] Vörös K., Farkas R. (2015). A kutyák szívférgessége. Kamarai Állatorvos.

[11] Jerzsele Á., Kovács D., Fábián P., Fehérvári P., Paszerbovics B., Bali K., Kaszab E., Mayer N., Karancsi Z. (2025). New Insights into the Prevalence of *Dirofilaria immitis* in Hungary. Animals.

[12] Széll Z., Sréter T., Csikós K., Kátai Z., Dobos-Kovács M., Vetési F., Varga I. (1999). Autochton *Dirofilaria repens* fertőzöttség kutyákban. Magy. Állatorvosok Lapja.

[13] Jacsó O., Fok É. (2006). A kutyák és macskák Dirofilaria repens fertőzöttségének kimutatása laboratóriumi módszerekkel. Magy. Allatorvosok Lapja.

[14] Simón F., Siles-Lucas M., Morchón R., González-Miguel J., Mellado I., Carretón E., Montoya-Alonso J.A. (2012). Human and Animal Dirofilariasis: The Emergence of a Zoonotic Mosaic. Clin. Microbiol. Rev..

[15] Kemenesi G., Kurucz K., Kepner A., Dallos B., Oldal M., Herczeg R., Vajdovics P., Bányai K., Jakab F. (2015). Circulation of *Dirofilaria repens, Setaria tundra*, and *Onchocercidae* Species in Hungary during the Period 2011–2013. Vet. Parasitol..

[16] Raele D.A., Pugliese N., Galante D., Latorre L.M., Cafiero M.A. (2016). Development and Application of a Loop-Mediated Isothermal Amplification (LAMP) Approach for the Rapid Detection of *Dirofilaria repens* from Biological Samples. PLoS Negl. Trop. Dis..

[17] Vörös K. (2019). Állatorvosi belgyógyászat I.—A Kutyák és Macskák Betegségei.

[18] Komáromi R.G. (2022). Kutya Szívférgesség Előfordulásának Retrospektív Vizsgálata Egy Állatorvosi Rendelőben. Doctoral Dissertation.

[19] Genchi C., Kramer L. (2017). Subcutaneous Dirofilariosis (*Dirofilaria repens*): An Infection Spreading throughout the Old World. Parasit. Vectors.

[20] Gabrielli S., Mendoza-Roldan J.A., Napoli E., De Benedetto G., Liapis D.D., Cascio A., Basile A., Iatta R., Perles L., Pombi M. (2025). Human Exposure to *Dirofilaria immitis* Following a Canine Heartworm Disease Elimination Program in Linosa Island (Sicily, Italy). Acta Trop..

[21] Schatz C., Füßl M., Caf Y., Schmitz K., Kresse D., Ludwig W., Walochnik J., Knabl L. (2025). A Rare Case Report of a Human *Dirofilaria repens* Infection. Microorganisms.

[22] Nelson C.T., McCall J.W., Jones S., Moorhead A. (2020). Highlights of the Current Canine Guidelines for the Prevention, Diagnosis, and Management of Heartworm (Dirofilaria immitis) Infection in Dogs.

[23] Bowman D., Atkins C. (2009). Heartworm Biology, Treatment, and Control. Vet. Clin. N. Am. Small Anim. Pract..

[24] Becker Z., Holló N., Farkas R., Gyurkovszky M., Reiczigel J., Olaszy K., Vári Z., Vörös K. (2022). Serodiagnostic Difficulties and Possibilities of Heartworm Disease in Regions Where Both *Dirofilaria immitis* and *Dirofilaria repens* Infections Occur. Acta Vet. Hung..

[25] Aththanayaka A.M.M.T.B., Dayananda B.S.W.M.T.B., Ranasinghe H.A.K., Amarasinghe L.D. (2024). Evolution of Dirofilariasis Diagnostic Techniques from Traditional Morphological Analysis to Molecular-Based Techniques: A Comprehensive Review. Front. Parasitol..

[26] Pietrzak D., Łuczak J., Wiśniewski M. (2024). Detecting *Dirofilaria immitis*: Current Practices and Novel Diagnostic Methods. Pathogens.

[27] Venco L., Manzocchi S., Genchi M., Kramer L.H. (2017). Heat Treatment and False-Positive Heartworm Antigen Testing in Ex Vivo Parasites and Dogs Naturally Infected by *Dirofilaria repens* and *Angiostrongylus vasorum*. Parasit. Vectors.

[28] Laidoudi Y., Davoust B., Varloud M., Niang E.H.A., Fenollar F., Mediannikov O. (2020). Development of a Multiplex QPCR-Based Approach for the Diagnosis of *Dirofilaria immitis, D. repens* and *Acanthocheilonema reconditum*. Parasit. Vectors.

[29] Jaratsing P., Viseshakul N., Areekit S., Chansiri K. (2016). Comparative Studies on Nucleic Acid Based Biosensors for Identification of Filarial Nematode. J. Med. Assoc. Thail..

[30] Tahir D., Bittar F., Barré-Cardi H., Sow D., Dahmani M., Mediannikov O., Raoult D., Davoust B., Parola P. (2017). Molecular Survey of *Dirofilaria immitis* and *Dirofilaria repens* by New Real-Time TaqMan^®^ PCR Assay in Dogs and Mosquitoes (Diptera: *Culicidae*) in Corsica (France). Vet. Parasitol..

[31] Kumar B., Maharana B.R., Brahmbhatt N.N., Thakre B.J., Parmar V.L. (2021). Development of a Loop-Mediated Isothermal Amplification Assay Based on RoTat1.2 Gene for Detection of *Trypanosoma evansi* in Domesticated Animals. Parasitol. Res..

[32] Raele D.A., Pugliese N., La Bella G., Calvario A., Scarasciulli M., Vasco I., La Salandra G., Cafiero M.A. (2021). Case Report: Molecular Detection of *Dirofilaria repens* in an Italian Patient after a Stay in Tanzania. Am. J. Trop. Med. Hyg..

[33] Smith R., Tomlinson T., Bowles J., Starkey L. (2024). Comparative Performance Analysis of Different Microfilaria Testing Methods for *Dirofilaria immitis* in Canine Blood. Parasit. Vectors.

[34] Cho J., Jeong S.-Y., Kim M.-S., Cho W.S., Kim D.-W., Park C. (2024). Loop-Mediated Isothermal Amplification Polymerase Chain Reaction in Place of a Modified Knott Test in Screening Dogs for Heartworm (*Dirofilaria immitis*) Infection Combined with Antigen Detection Test. Am. J. Vet. Res..

[35] Mori Y., Kanda H., Notomi T. (2013). Loop-Mediated Isothermal Amplification (LAMP): Recent Progress in Research and Development. J. Infect. Chemother..

[36] Notomi T., Okayama H., Masubuchi H., Yonekawa T., Watanabe K., Amino N., Hase T. (2000). Loop-Mediated Isothermal Amplification of DNA. Nucleic Acids Res..

[37] Mbithi A., Kusza S., Bagi Z. (2024). A Review of Loop Mediated Isothermal Amplification in Pathogen Detection: Pros and Cons. Állatteny. takarm..

[38] Zhou D., Guo J., Xu L., Gao S., Lin Q., Wu Q., Wu L., Que Y. (2014). Establishment and Application of a Loop-Mediated Isothermal Amplification (LAMP) System for Detection of Cry1Ac Transgenic Sugarcane. Sci. Rep..

[39] Khamlor T., Pongpiachan P., Parnpai R., Punyawai K., Sangsritavong S., Chokesajjawatee N. (2015). Bovine Embryo Sex Determination by Multiplex Loop-Mediated Isothermal Amplification. Theriogenology.

[40] Dini P., Van Poucke M., Herrera C., Peelman L., Daels P. (2016). Preimplantation Gender Determination on Equine Embryos Using LAMP. J. Equine Vet. Sci..

[41] Wong Y.-P., Othman S., Lau Y.-L., Radu S., Chee H.-Y. (2018). Loop-mediated Isothermal Amplification (LAMP): A Versatile Technique for Detection of Micro-organisms. J. Appl. Microbiol..

[42] Zhang X., Liao M., Jiao P., Luo K., Zhang H., Ren T., Zhang G., Xu C., Xin C., Cao W. (2010). Development of a Loop-Mediated Isothermal Amplification Assay for Rapid Detection of Subgroup J Avian Leukosis Virus. J. Clin. Microbiol..

[43] Tran D.H., Tran H.T., Vo B., Than T.T., Nguyen V.T., Le V.P., Phung H. (2024). Enhancing Classical Swine Fever Virus Identification: The Advantages of Field-LAMP Testing. Aust. Vet. J..

[44] Longhi S.A., García Casares L.J., Muñoz-Calderón A.A., Alonso-Padilla J., Schijman A.G. (2023). Combination of Ultra-Rapid DNA Purification (PURE) and Loop-Mediated Isothermal Amplification (LAMP) for Rapid Detection of *Trypanosoma cruzi* DNA in Dried Blood Spots. PLoS Negl. Trop. Dis..

[45] Rako L., Agarwal A., Semeraro L., Broadley A., Rodoni B.C., Blacket M.J. (2021). A LAMP (Loop-mediated Isothermal Amplification) Test for Rapid Identification of Khapra Beetle (*Trogoderma granarium*). Pest Manag. Sci..

[46] Lai M.-Y., Abdul Hamid M.H., Jelip J., Mudin R.N., Lau Y.-L. (2023). Evaluation of A Simple DNA Extraction Method and Its Combination with Loop-Mediated Isothermal Amplification Assays for Rapid Plasmodium Knowlesi Diagnosis. Trop. Med. Infect. Dis..

[47] Bhadra S., Pothukuchy A., Shroff R., Cole A.W., Byrom M., Ellefson J.W., Gollihar J.D., Ellington A.D. (2018). Cellular Reagents for Diagnostics and Synthetic Biology. PLoS ONE.

[48] Quoc N.B., Phuong N.D.N., Chau N.N.B., Linh D.T.P. (2018). Closed Tube Loop-Mediated Isothermal Amplification Assay for Rapid Detection of Hepatitis B Virus in Human Blood. Heliyon.

[49] Kumar T.S., Radhika K., Joseph Sahaya Rajan J., Makesh M., Alavandi S.V., Vijayan K.K. (2021). Closed-Tube Field-Deployable Loop-Mediated Isothermal Amplification (LAMP) Assay Based on Spore Wall Protein (SWP) for the Visual Detection of *Enterocytozoon hepatopenaei* (EHP). J. Invertebr. Pathol..

[50] Centeno-Cuadros A., Abbasi I., Nathan R. (2017). Sex Determination in the Wild: A Field Application of Loop-Mediated Isothermal Amplification Successfully Determines Sex across Three Raptor Species. Mol. Ecol. Resour..

[51] Lee P.L.M. (2017). DNA Amplification in the Field: Move over PCR, Here Comes LAMP. Mol. Ecol. Resour..

[52] Khangembam R., Tóth M., Vass N., Várady M., Czeglédi L., Farkas R., Antonopoulos A. (2021). Point of Care Colourimetric and Lateral Flow LAMP Assay for the Detection of Haemonchus Contortus in Ruminant Faecal Samples. Parasite.

[53] Moehling T., Choi G., Dugan L., Salit M., Meagher R. (2021). LAMP Diagnostics at the Point-of-Care: Emerging Trends and Perspectives for the Developer Community. Expert Rev. Mol. Diagn..

[54] Park J.W. (2022). Principles and Applications of Loop-Mediated Isothermal Amplification to Point-of-Care Tests. Biosensors.

[55] Bujtor Z.K., Zenke P. (2026). A LAMP- (hurok által közvetített izotermikus sokszorosítás) technika tesztelése kutyák szívférgességének (*Dirofilaria immitis*) kimutatására = Testing the LAMP (loop-mediated isothermal amplification) technique for the detection of heartworm disease (*Dirofilaria immitis*) in dogs. Magy. Állatorvosok Lapja.

[56] Tamura K., Stecher G., Peterson D., Filipski A., Kumar S. (2013). MEGA6: Molecular Evolutionary Genetics Analysis Version 6.0. Mol. Biol. Evol..

[57] Samal K., Sahoo J., Behera L., Dash T. (2021). Understanding the BLAST (Basic Local Alignment Search Tool) Program and a Step-by-Step Guide for Its Use in Life Science Research. Bhartiya Krishi Anusandhan Patrika.

[58] Clopper C.J., Pearson E.S. (1934). The use of confidence or fiducial limits illustrated in the case of binomial. Biometrika.

[59] Nagamine K., Hase T., Notomi T. (2002). Accelerated Reaction by Loop-Mediated Isothermal Amplification Using Loop Primers. Mol. Cell. Probes.

[60] Tomita N., Mori Y., Kanda H., Notomi T. (2008). Loop-Mediated Isothermal Amplification (LAMP) of Gene Sequences and Simple Visual Detection of Products. Nat. Protoc..

[61] Parida M., Sannarangaiah S., Dash P.K., Rao P.V.L., Morita K. (2008). Loop Mediated Isothermal Amplification (LAMP): A New Generation of Innovative Gene Amplification Technique; Perspectives in Clinical Diagnosis of Infectious Diseases. Rev. Med. Virol..

[62] Njiru Z.K. (2012). Loop-Mediated Isothermal Amplification Technology: Towards Point of Care Diagnostics. PLoS Negl. Trop. Dis..

[63] Mori Y., Nagamine K., Tomita N., Notomi T. (2001). Detection of Loop-Mediated Isothermal Amplification Reaction by Turbidity Derived from Magnesium Pyrophosphate Formation. Biochem. Biophys. Res. Commun..

[64] Goto M., Honda E., Ogura A., Nomoto A., Hanaki K.-I. (2009). Colorimetric Detection of Loop-Mediated Isothermal Amplification Reaction by Using Hydroxy Naphthol Blue. BioTechniques.

[65] Tanner N.A., Zhang Y., Evans T.C. (2015). Visual Detection of Isothermal Nucleic Acid Amplification Using PH-Sensitive Dyes. BioTechniques.

[66] Hayashida K., Kajino K., Hachaambwa L., Namangala B., Sugimoto C. (2015). Direct Blood Dry LAMP: A Rapid, Stable, and Easy Diagnostic Tool for Human African Trypanosomiasis. PLoS Negl. Trop. Dis..

[67] Mgawe C., Shilluli C., Nyanjom S., Odari E., Linnes J.C., Kanoi B.N., Gitaka J., Ochola L. (2023). Application of Multiple Binding Sites for LAMP Primers across *P. falciparum* Genome Improves Detection of the Parasite from Whole Blood Samples. Front. Malar..

[68] Sriworarat C., Phumee A., Mungthin M., Leelayoova S., Siriyasatien P. (2015). Development of Loop-Mediated Isothermal Amplification (LAMP) for Simple Detection of Leishmania Infection. Parasit. Vectors.

[69] Gummery L., Jallow S., Raftery A.G., Bennet E., Rodgers J., Sutton D.G.M. (2020). Comparison of Loop-Mediated Isothermal Amplification (LAMP) and PCR for the Diagnosis of Infection with *Trypanosoma brucei* ssp. in Equids in The Gambia. PLoS ONE.

[70] Aydin-Schmidt B., Xu W., González I.J., Polley S.D., Bell D., Shakely D., Msellem M.I., Björkman A., Mårtensson A. (2014). Loop Mediated Isothermal Amplification (LAMP) Accurately Detects Malaria DNA from Filter Paper Blood Samples of Low Density Parasitaemias. PLoS ONE.

[71] Zorkóczy O.K., Gyurcsó A., Ózsvári L., Lehotzky P., Sanil R., Zenke P. (2024). Development of a Loop-Mediated Isothermal Amplification Technique for Sex Detection in Cervidae Species. Mamm. Biol..

[72] Vincent J.P., Existe A.V., Komaki-Yasuda K., Boncy J., Kano S. (2023). Performance of the Procedure for Ultra-Rapid Extraction and Loop-Mediated Isothermal Amplification (PURE-LAMP) Method to Detect Malaria in Haiti. Infect. Dis. Poverty.

[73] Khan M., Wang R., Li B., Liu P., Weng Q., Chen Q. (2018). Comparative Evaluation of the LAMP Assay and PCR-Based Assays for the Rapid Detection of Alternaria Solani. Front. Microbiol..

[74] Ahmadi Y., Yu Y., Cui Z., Huang W., Andersson M. (2025). Loop-Mediated Isothermal Amplification (LAMP) for the Diagnosis of Sexually Transmitted Infections: A Review. Microb. Biotechnol..

[75] Augustine R., Hasan A., Das S., Ahmed R., Mori Y., Notomi T., Kevadiya B., Thakor A. (2020). Loop-Mediated Isothermal Amplification (LAMP): A Rapid, Sensitive, Specific, and Cost-Effective Point-of-Care Test for Coronaviruses in the Context of COVID-19 Pandemic. Biology.

[76] Tanner N., Zhang Y., Evans T. (2012). Simultaneous Multiple Target Detection in Real-Time Loop-Mediated Isothermal Amplification. BioTechniques.

[77] Jiang Y., Bhadra S., Li B., Wu Y., Milligan J., Ellington A. (2015). Robust Strand Exchange Reactions for the Sequence-Specific, Real-Time Detection of Nucleic Acid Amplicons. Anal. Chem..

[78] Hardinge P., Murray J. (2019). Reduced False Positives and Improved Reporting of Loop-Mediated Isothermal Amplification Using Quenched Fluorescent Primers. Sci. Rep..

