# Supplementary material for: Rapid Point-of-Care Detection of Dirofilaria immitis and Dirofilaria repens in Canine Blood Using Two Direct Closed-Tube LAMP Assays
Source: Animals (Basel). 2026 Jun 12;16(12):1820. doi: 10.3390/ani16121820 (PMC13295366; doi:10.3390/ani16121820)
Supplement: Supplementary file 1 [file animals-16-01820-s001.zip › animals-4327794-supplementary.pdf]

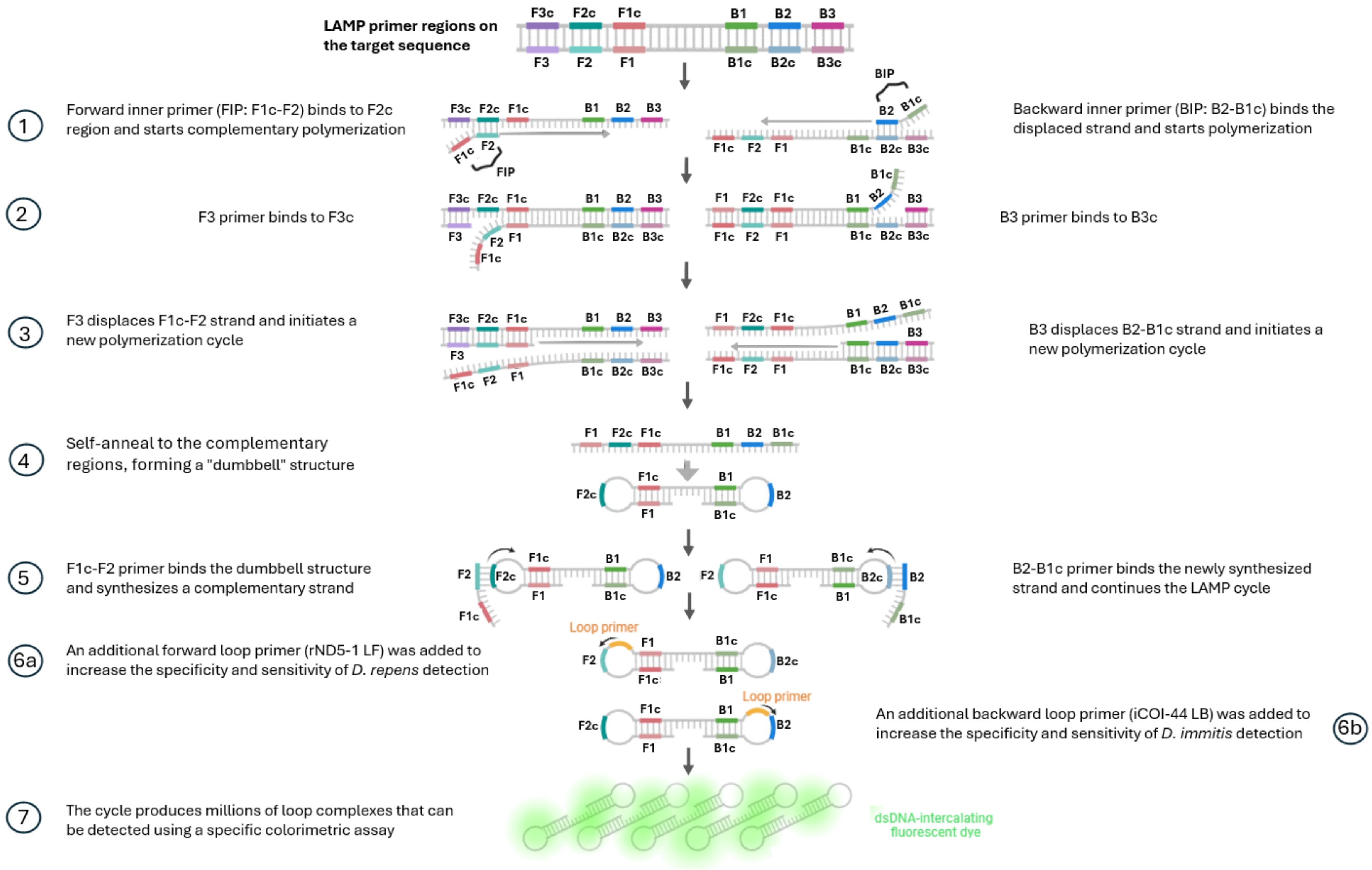


**Figure S1.** Schematic representation of the loop-mediated isothermal amplification (LAMP) mechanism and primer binding sites on the target DNA sequence. The inner primers (FIP: F1c–F2 and BIP: B2–B1c) initiate strand synthesis, while the outer primers (F3 and B3) displace the newly synthesized strands, leading to the formation of a characteristic dumbbell-shaped DNA structure. This structure serves as the starting template for cyclic amplification, generating stem-loop DNA products through continuous strand displacement. Additional loop primers accelerate the amplification reaction and improve assay sensitivity and specificity. In this study, species-specific primer sets targeting the mitochondrial COI gene of *Dirofilaria immitis* and the ND5 gene of *D. repens* were applied for direct LAMP detection from canine blood samples. The accumulation of amplified double-stranded DNA can be visualized using a dsDNA-intercalating fluorescent dye.

**Table S1.** LAMP primer sets designed and analyzed in silico for the detection of *Dirofilaria (D.) immitis*. Nucleotides highlighted in red indicate mismatches compared with the corresponding *D. repens* sequences.

| Primers | **iCOI-1** - 14 base differences from *D. repens* |
|---|---|
| F3 | GCCTTTGATATTGGGTGC |
| B3 | AAGATAACTCAGGCTGACC |
| FIP | TCAACAACGCAACAAAAGTAATCC-CCCTGAAATGGCTTTTCC |
| BIP | ATCAATCTTTTTTTATTGGGGGGGG-CTCTACACTCAAAGGAGGAT |

| Primers | **iCOI-44** - 15 base differences from *D. repens* |
|---|---|
| F3 | TTTTGGACATCCTGAGGTT |
| B3 | GCAGCACTAAAATAAGTACGA |
| FIP | GGCCAAACAAACGATCCTTATCAG-TTATTTTACCGGTGTTTGGGAT |
| BIP | TGACTTTTGCTTCTATTTGGATTGC-GTATCAATATCCAAACCAGCT |
| LB | TATTGGGGACTTCTGTTTGGGGT |

| Primers | **iCOI-2*** - 11 base differences from *D. repens* |
|---|---|
| F3 | ATGCCTATTTTGATTGGTGG |
| B3 | CTCTACACTCAAAGGAGGAT |
| FIP | GCATTAACACGAGGAAAAGCCATTT-TTTTGGTAATTGGATGTTGCC |
| BIP | GTTGCGTTGTTGATGGTTTATCAA-AAGTTCAACTACTCCCAGG |
| LF | CAGGGGCACCCAATATCAA |

| Primers | **iND1-1** - 6 base differences from *D. repens* |
|---|---|
| F3 | TTTTATTTTGCAGTCTGTTGC |
| B3 | CAAACCCACAAACAGGAAC |
| FIP | ACCTTATTAGGACCAATTCGACACT-TTTTTTGACTTTGTTGGAGCG |
| BIP | ATGGTTTGAAGTTGTTGAAAAAGGA-CAAAAAAGAAATCCAACTAGAGCA |

| Primers | **iCOI-15** - 16 base differences from *D. repens* |
|---|---|
| F3 | GGTAATTGGATGTTGCCTTTG |
| B3 | GACCTAAAATCATTCTATCCAAAG |
| FIP | CCATCAACAACGCAACAAAAGTAAT-CTGAAATGGCTTTTCCTCG |
| BIP | CAATCTTTTTTTATTGGGGGGGT-GCTGACCCTCTACACTCAA |

| Primers | **iND1-11** - 8 base differences from *D. repens* |
|---|---|
| F3 | TCTGTTGCTTTTTTGACTTTG |
| B3 | CAAACCCACAAACAGGAAC |
| FIP | ACCACAATAAGCAACCTTATTAGGA-TTGGAGCGTCATTTTTTAGGT |
| BIP | ATGGTTTGAAGTTGTTGAAAAAGGA-CAAAAAAGAAATCCAACTAGAGCA |

| Primers | **iCOI-32** - 17 base differences from *D. repens* |
|---|---|
| F3 | GCTTTTCCTCGTGTTAATGC |
| B3 | CAGTAACCATAAAATTAATAGCACC |
| FIP | TCAACTACTCCCAGGACCCC-TACTTTTGTTGCGTTGTTGAT |
| BIP | TTATCCTCCTTTGAGTGTAGAGGG-ATACCAACAGTATGAAGACCTA |
| LB | TCAGCCTGAGTTATCTTTGGATAGA |

| Primers | **iND1-22**- 9 base differences from *D. repens* |
|---|---|
| F3 | TGACTTTGTTGGAGCGTC |
| B3 | AACCCACAAACAGGAACA |
| FIP | AGCTTGAAAAAAACCACAATAAGCA-ATTTTTTAGGTGGTTCTCAGTG |
| BIP | ATGGTTTGAAGTTGTTGAAAAAGGA-CAAAAAAGAAATCCAACTAGAGCA |

*: the primer sequences are identical to those reported by Cho et al. (2024); however, in that study, the set was supplemented with an additional LB loop primer [34]

**Table S2.** LAMP primer sets designed and analyzed in silico for the detection of *Dirofilaria (D.) repens*. Nucleotides highlighted in red indicate mismatches compared with the corresponding *D. immitis* sequences.

| Primers | rCOI-2* - min. 8 base differences from *D. immitis* |
|---|---|
| F3 | GCGTTTCCTCGTGTTAATGC |
| B3 | AACCATAAAATTAATAGCACCCAAC |
| FIP | CCAGGTCCACCACCAATAAAAAAAG-TGTCTTTTTGGTTTACTTTTGTTGC |
| BIP | TCCTCCTTTAAGTGTTGATGGTCAA-CCAATACCTACAGTATGTAAACCCA |

| Primers | rCOI-27 - 13 base differences from *D. immitis* |
|---|---|
| F3 | GCCTGTTTTTGGTATTATTAGTGAA |
| B3 | AAAATCTTAACAGCTCTAGGAAT |
| FIP | ACAGAAGTACCTAAAACAGCAATCC-CTGATAAGGATCGTTTGTTTGG |
| BIP | GAGGTCATCATATGTATACTGCTGG-CAATAATCATAGTAGCAGCACT |

| Primers | rCOI-4 - 15 base differences from *D. immitis* |
|---|---|
| F3 | GGTTTACTTTTGTTGCTTTGT |
| B3 | ACTAATCTGATCTAAAGTAACAGC |
| FIP | GACCATCAACACTTAAAGGAGGATA-AATCTTTTTTTATTGGTGGTGGA |
| BIP | ACTGTAGGTATTGGTTCTTTGTTGG-AGTAGAACGCATATTCTGAGT |

| Primers | rCOI-36 - 9 base differences from *D. immitis* |
|---|---|
| F3 | TTTTTGACTGATAAGGATCGTT |
| B3 | ACTATAAGTTCAACATCAAAGAGG |
| FIP | TGATGACCTCAAACAGAAGTACCTA-GTCAGACTAGTATAACTTTTGCT |
| BIP | TCGAACTTATTTTAGTGCTGCTACT-CCAAAAAAAGTACCCAACCA |

| Primers | rCOI-14 - 11 base differences from *D. immitis* |
|---|---|
| F3 | GAGGTTTATGTTATTATTTTGCCTG |
| B3 | ACAGCTCTAGGAATAGCAATA |
| FIP | CAGCAATCCAAATAGAAGCAAAAGT-GTGAATGTGTTTTGTTTTTGACTG |
| BIP | TTAGGTACTTCTGTTTGAGGTCATC-CAGCACTAAAATAAGTTCGAGTA |
| LF | CCTAGCAAACAAACCAGTCTGATCA |

| Primers | rCOI44 - 15 base differences from *D. immitis* |
|---|---|
| F3 | TTTTGGTCATCCTGAGGTT |
| B3 | GCAGCACTAAAATAAGTTCGA |
| FIP | GACCAAACAAACGATCCTTATCAG-TTATTTTGCCTGTTTTTGGTAT |
| BIP | TAACTTTTGCTTCTATTTGGATTGC-GTATCAATATCTAAACCAGCA |
| LB | TTTTAGGTACTTCTGTTTGAGGC |

| Primers | rCOI-18 - 13 base differences from *D. immitis* |
|---|---|
| F3 | GCCTGTTTTTGGTATTATTAGTGAA |
| B3 | ACAGCTCTAGGAATAGCAATA |
| FIP | CAGCAATCCAAATAGAAGCAAAAGT-TTTGTTTTTGACTGATAAGGATCG |
| BIP | TTAGGTACTTCTGTTTGAGGTCATC-CAGCACTAAAATAAGTTCGAGTA |

| Primers | rND5-1- 17 base differences from *D. immitis* |
|---|---|
| F3 | CTTTTGTTAAGGGTGGTCAG |
| B3 | CAAAAACCAGAAAAAACCAAAGT |
| FIP | ACTATGAACCAAACAACTAACAGGA-TATCCTTTTGGTAGTTGGCTT |
| BIP | GTTACTGCTGGTGTTATGTTAATGG-ACAAAAGACAACACATCAGAA |
| LF | GGGGCAGCCATAGCTTTAGG |

*: the primer sequences are identical to those reported by Raele et al. (2016) [16]

**Table S3.** Results of in vitro testing of fourteen newly designed and two previously published (iCOI-2 and rCOI-2) LAMP primer sets for the detection of *Dirofilaria immitis* and *D. repens* using DNA isolates ($n$ = 40) from canine blood samples. Corresponding Ct values obtained by duplex quantitative real-time PCR (qPCR) are also shown. Ct: cycle threshold; D: *Dirofilaria*; +: positive reaction; −: negative reaction. Background shading indicates false-positive or false-negative results. *: the primer sequences are identical to those reported by Cho et al. (2024); however, in that study, the set was supplemented with an additional LB loop primer [34]. **: the primer sequences are identical to those reported by Raele et al. (2016) [16]

| Sample ID | qPCR Ct | | *Dirofilaria immitis* primer sets | | | | | | | | *Dirofilaria repens* primer sets | | | | | | | |
|---|---|---|---|---|---|---|---|---|---|---|---|---|---|---|---|---|---|---|
| | *D. immitis* | *D. repens* | iCOI-1 | iCOI-2* | iCOI-15 | iCOI-32 | iCOI-44 | iND1-1 | iND1-11 | iND1-22 | rCOI-2** | rCOI-4 | rCOI-14 | rCOI-18 | rCOI-27 | rCOI-36 | rCOI-44 | rND5-1 |
| Di1 | 27.80 | neg | + | + | - | - | + | + | + | + | - | - | - | + | - | - | - | - |
| Di2 | 32.70 | neg | + | + | - | + | + | + | + | + | - | - | - | - | - | - | - | - |
| Di3 | 29.50 | neg | + | + | - | + | + | + | + | + | - | - | - | - | - | - | - | - |
| Di4 | 26.10 | neg | + | + | - | + | + | + | + | + | - | - | - | - | - | + | - | - |
| Di5 | 25.50 | neg | + | + | - | + | + | + | + | + | - | - | - | - | - | + | - | - |
| Di6 | 27.50 | neg | + | + | - | + | + | + | + | + | - | - | - | - | - | - | - | - |
| Di7 | 23.20 | neg | + | + | + | + | + | + | + | + | - | - | + | + | - | - | - | - |
| Di8 | 21.90 | neg | + | + | + | + | + | + | + | + | - | - | + | - | - | + | + | - |
| Di9 | 25.60 | neg | + | + | - | + | + | + | + | + | - | - | + | - | - | + | - | - |
| Di10 | 23.90 | neg | + | + | - | + | + | + | + | + | - | - | + | - | - | - | - | - |
| Di11 | 24.20 | neg | + | + | - | + | + | + | + | + | - | - | + | - | - | - | - | - |
| Di12 | 24.30 | neg | + | + | - | + | + | + | + | + | - | - | + | - | - | + | - | - |
| Dr1 | neg | 25.80 | - | - | - | - | - | - | - | - | + | + | + | + | - | - | - | + |
| Dr2 | neg | 26.83 | - | - | - | - | - | - | - | - | + | + | + | + | - | - | - | + |
| Dr3 | neg | 21.68 | - | + | - | - | - | - | - | - | + | + | + | - | - | - | - | + |
| Dr4 | neg | 23.48 | - | - | - | - | - | - | - | - | + | + | + | - | - | - | - | + |
| Dr5 | neg | 24.58 | - | - | - | - | - | - | - | - | + | + | + | - | - | - | - | + |
| Dr6 | neg | 26.19 | - | - | - | - | - | - | - | - | + | + | + | - | - | + | - | + |
| Dr7 | neg | 24.69 | - | - | - | - | - | - | - | - | + | + | + | + | - | - | - | + |
| Dr8 | neg | 22.60 | - | + | - | - | - | - | - | - | + | + | + | - | - | - | - | + |
| Dr9 | neg | 20.95 | - | + | - | - | - | - | - | - | + | + | + | + | - | + | - | + |
| Dr10 | neg | 21.33 | - | - | - | - | - | - | - | - | + | + | + | - | - | + | - | + |
| Dr11 | neg | 26.78 | - | - | - | - | - | - | - | - | + | + | + | - | - | - | - | + |
| Dr12 | neg | 20.27 | - | + | - | - | - | - | - | - | + | + | + | + | - | - | - | + |
| Dir1 | 24.30 | 34.63 | + | + | - | + | + | + | + | + | + | + | + | + | - | - | - | + |
| Dir2 | 25.89 | 35.28 | + | + | - | + | + | + | + | + | + | + | + | - | - | - | - | + |
| Dir3 | 20.00 | 32.91 | + | + | + | + | + | + | + | + | + | + | + | + | - | - | - | + |
| Dir4 | 23.10 | 22.08 | + | + | - | + | + | + | + | + | + | + | + | + | - | + | - | + |
| Dir5 | 23.70 | 31.52 | + | + | - | + | + | + | + | + | + | + | + | + | - | - | - | + |
| Dir6 | 25.20 | 27.72 | + | + | - | + | + | + | + | + | + | + | + | + | - | - | - | + |
| Dir7 | 20.50 | 37.10 | + | + | - | + | + | + | + | + | + | + | + | + | - | - | - | + |
| D1 | neg | neg | - | - | - | - | - | - | - | - | - | - | - | - | - | - | - | - |
| D2 | neg | neg | - | - | - | - | - | - | - | - | - | - | - | - | - | - | - | - |
| D3 | neg | neg | - | - | - | - | - | - | - | - | - | - | - | - | - | - | - | - |
| D4 | neg | neg | - | - | - | - | - | - | - | - | - | - | - | - | - | - | - | - |
| D5 | neg | neg | - | - | - | - | - | - | - | - | - | - | - | - | - | - | - | - |
| D6 | neg | neg | - | - | - | - | - | - | - | - | - | - | - | - | - | - | - | - |
| D7 | neg | neg | - | - | - | - | - | - | - | - | - | - | - | - | - | - | - | - |
| D8 | neg | neg | - | - | - | - | - | - | - | - | - | - | - | - | - | - | - | - |
| D9 | neg | neg | - | - | - | - | - | - | - | - | - | - | - | - | - | - | - | - |

**Table S4.** Sample-level and reaction-level concordance of purified DNA and direct blood LAMP assays relative to qPCR results. Partial concordance indicates co-infected samples in which only one target species was correctly detected by LAMP.

| Analysis level | Target/sample classification | Correct | Partial concordance | Incorrect |
|---|---|---|---|---|
| Sample-level (n = 90) | Purified DNA | 87 | 1 | 2 |
| | Direct blood LAMP | 79 | 4 | 7 |
| Reaction-level (n = 180) | Purified DNA *D. immitis* | 87/90 | NA | 3/90 |
| | Direct blood *D. immitis* | 85/90 | NA | 5/90 |
| | Purified DNA *D. repens* | 90/90 | NA | 0/90 |
| | Direct blood *D. repens* | 83/90 | NA | 7/90 |

**Table S5.** Diagnostic performance of the selected LAMP assays (iCOI-44 for *Dirofilaria immitis* and rND5-1 for *Dirofilaria repens*) in the independent 50-DNA sample validation cohort. Relative sensitivity, specificity, and accuracy were calculated using qPCR results as the reference comparator. TP, true positive; TN, true negative; FP, false positive; FN, false negative; CI, confidence interval. Ninety-five percent confidence intervals (95% CI) were calculated using the Clopper–Pearson exact binomial method.

| Assay | Samples | TP | TN | FP | FN | Relative sensitivity (95% CI) | Relative specificity (95% CI) | Relative accuracy (95% CI) |
|---|---|---|---|---|---|---|---|---|
| *D. immitis* (iCOI-44) | 50 | 33 | 14 | 0 | 3 | 91.7% (77.5–98.2) | 100% (76.8–100) | 94.0% (83.5–98.8) |
| *D. repens* (rND5-1) | 50 | 12 | 38 | 0 | 0 | 100% (73.5–100) | 100% (90.8–100) | 100% (92.9–100) |
